# Physicians’ knowledge on specific rare diseases and its associated factors: a national cross-sectional study from China

**DOI:** 10.1186/s13023-022-02243-7

**Published:** 2022-03-05

**Authors:** Huanyu Zhang, Ying Xiao, Xinyue Zhao, Zhuang Tian, Shu-yang Zhang, Dong Dong

**Affiliations:** 1 Shenzhen Research Institute, The Chinese University of Hong Kong, Shenzhen, 518000 China; 2 Department of Cardiology, Peking Union Medical College Hospital, Chinese Academy of Medical Sciences and Peking Union Medical College, Beijing, 100730 China; 3 JC School of Public Health and Primary Care, Faculty of Medicine, The Chinese University of Hong Kong, Shatin, Hong Kong SAR, China

**Keywords:** Physicians, Rare diseases, Knowledge, China, Patient organizations

## Abstract

**Background:**

Rare disease patients often experience diagnosis delays or misdiagnosis, which may be due to lack of knowledge on rare diseases among physicians.

**Objective:**

To assess Chinese physicians’ knowledge on specific rare diseases and identify its associated factors.

**Methods:**

Thirty-four patient organizations with a unique disease of interest were invited to develop 3 knowledge questions for each rare disease to assess physicians’ knowledge on the disease that they felt most experienced in. The total knowledge score for each participant ranged from a score of 0 to 3. A national cross-sectional study conducted in a cohort of 3197 physicians from 6 provinces across western, central and eastern China. The demographic information of the participants was collected including gender, age, birthplace, income, education, hospital class, working title, working years, and specialty. A multiple linear regression analysis was performed to assess the independent associations between the physician variables and the total knowledge score.

**Results:**

Two thousand, one hundred and fifteen (66.16%) of the involved physicians obtained a total knowledge score of 2 or 3. The median knowledge scores of 10 (29.4%) rare diseases were a score of 1.5 or below. Physicians with female gender (β = 0.08, *p* < 0.05 for females vs. males), and a monthly income of 5000–10,000 RMB (β = 0.11, *p* < 0.01 for 5000–10,000 vs. < 5000) and 10,000–30,000 RMB (β = 0.14, *p* < 0.05) were associated with a higher score. Specialties of physicians who received a relatively higher score included internal medicine, obstetrics and gynecology, radiology, intensive care unit, and surgery.

**Conclusions:**

Almost two thirds of the participants had an average or good level of knowledge on the specific rare disease that they felt most experienced in. Physicians with female gender, a monthly income of 5000–10,000 RMB and 10,000–30000 RMB, and specialties of internal medicine, obstetrics and gynecology, radiology, intensive care unit, and surgery, were associated with a relatively higher knowledge score.

**Supplementary Information:**

The online version contains supplementary material available at 10.1186/s13023-022-02243-7.

## Background

Rare diseases are generally defined as serious, chronic, and often life-threatening conditions which affect a small percentage of population [1]. Among the 6000–8000 rare diseases that have been identified worldwide, 6–8% of the global population is affected [2, 3]. Although each rare disease represents varied problems experienced by patients, families, and caregivers, one of the most common issues that prevents patients from achieving a better quality of life is difficulty in diagnosis [4–6]. A survey published in 2013 revealed that it took an average of 7.6 years in the United States and 5.6 years in the United Kingdom for rare disease patients to receive an appropriate diagnosis [7]. Misdiagnosis or diagnosis delays can lead to the deterioration of symptoms and disease progression to rare disease patients, resulting in inappropriate medical interventions and additional medical costs [4, 5, 8]. This situation is not only frustrating to patients and health care professionals, but also challenging to the healthcare systems around the world.

In a traditional patient-physician relationship, physicians often dominate in the medical encounters assuming they have sufficient (and superior) knowledge and skills regarding the diagnosis and treatment. However, in the context of rare diseases, due to low prevalence and high clinical complexity, physicians often lack knowledge and experience, resulting in a shift in the traditional patient-physician interaction [9]. Rare disease patients and their families have to become knowledgeable about their own diseases and educate physicians about their conditions [4, 5, 9]. This role discrepancy may cause further problems. A number of studies have shown that lack of rare disease awareness and knowledge among physicians could lead to delayed diagnosis or misdiagnosis [4, 5, 9–11]. A previous study indicated that 78.8% of patients affected by rare diseases have not received proper care because of limited training of health care professionals in this field [12].

In China, no epidemiological data on the prevalence of rare diseases is available so far. A recent study conducted in Hong Kong showed that the prevalence of rare diseases was estimated at 1 in 67 in Hong Kong population [13]. Hence, we estimated that approximately 21 million people are affected by rare diseases in China; this number represents a high probability of encountering such patients for health care professionals during their career. Efforts have been made to improve the quality of care for rare disease patients in China since 1980s [14]. In 2018, the first official list of rare diseases in China has been released [15]. Subsequently, a guideline for the diagnosis and treatment of 121 rare diseases defined by the list has been initiated to support clinical practice at the national level. However, difficulty in diagnosis among rare disease patients can still be observed in China. A recent national survey reported that 73% of the investigated rare disease patients were misdiagnosed; they had to spend an average of 4.3 years and visit 3 hospitals to access the correct diagnosis [6]. Additionally, due to the uneven distribution of healthcare resources in China, rare disease patients and their families often have to travel across provinces to obtain an accurate diagnosis, which could increase financial burden on the family [6, 16]. Although a few studies conducted in China have highlighted the importance of improving rare disease awareness and knowledge among physicians [6, 15], no disease-specific information about these unmet needs is available in China to the best of our knowledge. Hence, this study aimed to assess physicians’ knowledge on specific rare diseases and identify its associated factors at the national level in China.

## Methods

### Development of knowledge questions on rare diseases

In this study, we invited patient organizations with a specific disease of interest in China to develop questions so as to assess the degree of physicians’ knowledge on rare diseases. The following were the inclusion criteria for patient organizations: (1) the disease of interest of patient organizations is included in the official list of rare diseases in China; (2) an organizer is responsible for routine practice performed in the office or online chat groups regardless of official registration; (3) the patient organization with the largest number of patients or longest history will be selected if several patient organizations with the same disease of interest exist. After applying the inclusion criteria, we invited 50 patient organizations to participate in our study. Consequently, 34 patient organizations with a unique disease of interest agreed to be involved.

The process for developing knowledge questions on rare diseases started from May 2019. The involved patient organizations were required to provide three true–false questions regarding the knowledge on the disease of their interest, with the degree of difficulty ranging from low, medium, to high. A self-assessment on the quality of questions was conducted by the specialists acquainted by the patient organizations. After reviewing the contents and answers of the knowledge questions by researchers in this study, a total of 102 questions on 34 rare diseases were ready for further actions in December 2019 (Additional file 1).

### Study sample and data collection

A cross-sectional study was conducted in January 2020. Physicians from 6 provinces across eastern, central, and western China including Shandong, Jiangsu, Shanxi, Jiangxi, Sichuan, and Shannxi, were investigated. A non-random convenience sampling method was used to recruit physicians aged 18 years or above from around 200 hospitals located in the six provinces. Medical students who had not graduated were not included in the current study. The demographic information of the participants was collected including gender, age, birthplace, income, education, hospital class, working title, working years, and specialty. All the participants were anonymized and de-identified; any sensitive information on physicians was not accessible.

Regarding the medical encounter of rare diseases, the participants were investigated if they had experience of encountering a patient with any of 34 rare diseases of concern. Among the 4387 physicians who agreed to participate in the study, 1062 (24.2%) reported that they had no medical encounters of rare disease patients and 128 (2.9%) reported to have experience of encountering rare disease patients beyond the specific 34 rare diseases of interest; these physicians were excluded from the current study. Eligible participants were further asked to select one rare disease that they felt most experienced in and subsequently answer three knowledge questions regarding this rare disease. The knowledge questions were provided with three options, namely, true, false, and don’t know. If the participants didn’t choose the correct answer or choose ‘don’t know’ for each question, they would obtain a score of 0. Otherwise, they would obtain a score of 1. The total knowledge score for each participant ranged from a score of 0 to 3.

### Statistical analysis

Frequencies and percentages were calculated for descriptive data based on geographical locations. The median score with interquartile range (IQR) for each specific rare disease was reported. The characteristics of physicians with a total knowledge score ranging from a score of 0 to 3 as a categorical variable were compared. Fisher’s exact tests were used to determine the differences between the four groups of physicians. Univariable analyses were conducted to evaluate the relationship between the characteristics of physicians and the total knowledge score as a continuous variable. To assess the independent associations between the physician variables and the total knowledge score, a multiple linear regression analysis was performed. The variance inflation factor (VIF) for each independent variable in the regression model was detected to eliminate multicollinearity. A *p*-value lower than 0.05 was considered statistically significant. All the statistical analyses were conducted using R version 4.0.4.

## Results

Of the 4387 physicians who agreed to participate in this study, 3197 (72.9%) had experience of encountering patients with 34 rare diseases of concern. Among them, 54.5% were females, 44.5% aged 26–35 years, and 64.7% reported a master or higher degree. More detailed information on the demographics of the study sample is presented in Table 1.

**Table 1:** Demographic characteristics of physicians who had experience of encountering a patient with any of 34 rare diseases of concern

Characteristics	Total n = 3197	Eastern n = 1493	Central n = 1427	Western n = 277
	*Number (percent)*			
*Gender*				
Male	1456 (45.5%)	724 (48.5%)	620 (43.4%)	112 (40.4%)
Female	1741 (54.5%)	769 (51.5%)	807 (56.6%)	165 (59.6%)
*Age*				
18–25	41 (1.3%)	14 (0.9%)	22 (1.5%)	5 (1.8%)
26–35	1424 (44.5%)	695 (46.6%)	588 (41.2%)	141 (50.9%)
36–45	1075 (33.6%)	516 (34.6%)	484 (33.9%)	75 (27.1%)
46–55	544 (17.0%)	223 (14.9%)	276 (19.3%)	45 (16.2%)
56–65	106 (3.3%)	43 (2.9%)	53 (3.7%)	10 (3.6%)
66 +	7 (0.2%)	2 (0.1%)	4 (0.3%)	1 (0.4%)
*Birthplace*				
Urban area	2843 (88.9%)	1321 (88.5%)	1285 (90.0%)	237 (85.6%)
Rural area	351 (11.0%)	171 (11.5%)	140 (9.8%)	40 (14.4%)
Others	3 (0.1%)	1 (0.1%)	2 (0.1%)	0 (0.0%)
*Monthly income*				
< 5000	653 (20.4%)	118 (7.9%)	494 (34.6%)	41 (14.8%)
5000–10,000	2026 (63.4%)	995 (66.6%)	855 (59.9%)	176 (63.5%)
10,000–30,000	506 (15.8%)	376 (25.2%)	71 (5.0%)	59 (21.3%)
30,000–50,000	7 (0.2%)	2 (0.1%)	5 (0.4%)	0 (0.0%)
> 50,000	5 (0.2%)	2 (0.1%)	2 (0.1%)	1 (0.4%)
*Province*				
Shandong	298 (9.3%)	298 (20.0%)	0 (0.0%)	0 (0.0%)
Jiangsu	1195 (37.4%)	1195 (80.0%)	0 (0.0%)	0 (0.0%)
Jiangxi	468 (14.6%)	0 (0.0%)	468 (32.8%)	0 (0.0%)
Shanxi	959 (30.0%)	0 (0.0%)	959 (67.2%)	0 (0.0%)
Sichuan	178 (5.6%)	0 (0.0%)	0 (0.0%)	178 (64.3%)
Shannxi	99 (3.1%)	0 (0.0%)	0 (0.0%)	99 (35.7%)
*Education*				
Secondary vocational school	3 (0.1%)	0 (0.0%)	2 (0.1%)	1 (0.4%)
Three-year college	19 (0.6%)	3 (0.2%)	10 (0.7%)	6 (2.2%)
Bachelor’s degree	1104 (34.5%)	345 (23.1%)	667 (46.7%)	92 (33.2%)
Master’s degree	1782 (55.7%)	927 (62.1%)	727 (50.9%)	128 (46.2%)
Doctorate/postdoc	289 (9.0%)	218 (14.6%)	21 (1.5%)	50 (18.1%)
*Title*				
Primary	823 (25.7%)	334 (22.4%)	384 (26.9%)	105 (37.9%)
Middle	1208 (37.8%)	584 (39.1%)	544 (38.1%)	80 (28.9%)
Vice-senior	709 (22.2%)	350 (23.4%)	298 (20.9%)	61 (22.0%)
Senior	435 (13.6%)	217 (14.5%)	190 (13.3%)	28 (10.1%)
None	22 (0.7%)	8 (0.5%)	11 (0.8%)	3 (1.1%)
*Working years*				
≤ 5	828 (25.9%)	396 (26.5%)	348 (24.4%)	84 (30.3%)
6–10	779 (24.4%)	376 (25.2%)	327 (22.9%)	76 (27.4%)
11–15	514 (16.1%)	217 (14.5%)	254 (17.8%)	43 (15.5%)
16–20	368 (11.5%)	194 (13.0%)	156 (10.9%)	18 (6.5%)
21–25	333 (10.4%)	154 (10.3%)	154 (10.8%)	25 (9.0%)
26–30	216 (6.8%)	92 (6.2%)	105 (7.4%)	19 (6.9%)
30 +	159 (5.0%)	64 (4.3%)	83 (5.8%)	12 (4.3%)
*Hospital class*				
Tertiary hospital	3185 (99.6%)	1492 (99.9%)	1425 (99.9%)	268 (96.8%)
Secondary hospital	8 (0.3%)	1 (0.1%)	0 (0.0%)	7 (2.5%)
Primary hospital	4 (0.1%)	0 (0.0%)	2 (0.1%)	2 (0.7%)
*Specialty*				
Anesthesiology	33 (1.0%)	6 (0.4%)	27 (1.9%)	0 (0.0%)
Dermatology	57 (1.8%)	21 (1.4%)	32 (2.2%)	4 (1.4%)
Emergency medicine	89 (2.8%)	37 (2.5%)	52 (3.6%)	0 (0.0%)
Infectious diseases	69 (2.2%)	23 (1.5%)	44 (3.1%)	2 (0.7%)
Intensive care	86 (2.7%)	58 (3.9%)	24 (1.7%)	4 (1.4%)
Internal medicine	1370 (42.9%)	665 (44.5%)	549 (38.5%)	156 (56.3%)
Laboratory medicine	3 (0.1%)	0 (0.0%)	1 (0.1%)	2 (0.7%)
Obstetrics and gynecology	133 (4.2%)	62 (4.2%)	58 (4.1%)	13 (4.7%)
Oncology	74 (2.3%)	48 (3.2%)	21 (1.5%)	5 (1.8%)
Ophthalmology	69 (2.2%)	21 (1.4%)	48 (3.4%)	0 (0.0%)
Orthopedic surgery, medical cosmetology	11 (0.3%)	4 (0.3%)	7 (0.5%)	0 (0.0%)
Otolaryngology	37 (1.2%)	12 (0.8%)	21 (1.5%)	4 (1.4%)
Pain medicine	6 (0.2%)	0 (0.0%)	6 (0.4%)	0 (0.0%)
Pathology	8 (0.3%)	3 (0.2%)	5 (0.4%)	0 (0.0%)
Pediatrics	324 (10.1%)	138 (9.2%)	161 (11.3%)	25 (9.0%)
Psychiatry	17 (0.5%)	14 (0.9%)	3 (0.2%)	0 (0.0%)
Radiology	138 (4.3%)	69 (4.6%)	66 (4.6%)	3 (1.1%)
Sports medicine, rehabilitation	86 (2.7%)	26 (1.7%)	49 (3.4%)	11 (4.0%)
Stomatology	29 (0.9%)	5 (0.3%)	19 (1.3%)	5 (1.8%)
Surgery	415 (13.0%)	245 (16.4%)	151 (10.6%)	19 (6.9%)
Traditional Chinese medicine	39 (1.2%)	11 (0.7%)	25 (1.8%)	3 (1.1%)
Others	104 (3.3%)	25 (1.7%)	58 (4.1%)	21 (7.6%)

In Table 2, medians of the total knowledge score with IQRs for each rare disease were reported. Among the 34 rare diseases of concern, the most frequently chosen rare diseases that physicians felt experienced in were Albinism (14.58%), Hepatolenticular degeneration (Wilson’s Disease) (11.85%), Multiple Sclerosis (10.85%), and Hemophilia (9.16%). The median knowledge scores of 10 (29.4%) rare diseases were a score of 1.5 or below, including Tetrahydrobiopterin deficiency (1.00 ± 0.00), Spinal and bulbar muscular atrophy (1.00 ± 0.25), Type II glycogen storage disease (1.00 ± 0.50), Amyotrophic lateral sclerosis (1.00 ± 1.00), Osteogenesis imperfecta (1.00 ± 1.00), Prader–Willi syndrome (1.00 ± 1.00), Phenylketonuria (1.00 ± 1.00), Multiple sclerosis (1.00 ± 1.00), Spinocerebellar ataxia (1.00 ± 1.50), and Spinal muscular atrophy (1.50 ± 1.00).

**Table 2:** Medians of the total knowledge score by the 34 rare diseases of concern

Type of rare diseases	No. of physicians (%)	Total	Eastern	Central	Western
		*Median (IQR)*			
Albinism	466 (14.58%)	2.00 (2.00)	2.00 (2.00)	2.00 (2.00)	2.00 (1.00)
Hepatolenticular degeneration	379 (11.85%)	2.00 (1.00)	2.00 (1.00)	2.00 (1.00)	2.00 (1.00)
Multiple sclerosis	347 (10.85%)	1.00 (1.00)	1.00 (1.00)	1.00 (0.00)	1.00 (0.00)
Hemophilia	293 (9.16%)	3.00 (1.00)	3.00 (1.00)	3.00 (1.00)	3.00 (1.00)
Marfan syndrome	224 (7.01%)	2.00 (0.00)	2.00 (0.00)	2.00 (0.00)	2.00 (0.00)
Idiopathic pulmonary arterial hypertension	168 (5.25%)	2.00 (1.00)	2.00 (1.00)	2.00 (1.00)	2.00 (0.50)
Systemic sclerosis	152 (4.75%)	2.00 (1.00)	2.00 (1.00)	2.00 (1.00)	2.00 (1.25)
General myathenic gravis	146 (4.57%)	2.00 (2.00)	2.00 (1.00)	1.50 (1.75)	2.00 (2.00)
Amyotrophic lateral sclerosis	124 (3.88%)	1.00 (1.00)	1.00 (1.00)	1.00 (1.00)	2.00 (0.00)
Osteogenesis imperfecta	118 (3.69%)	1.00 (1.00)	1.00 (1.00)	1.50 (1.00)	2.50 (1.25)
Langerhans cell histiocytosis	110 (3.44%)	2.00 (1.00)	3.00 (1.00)	2.00 (1.00)	2.00 (1.00)
Neuromyelitis optica	89 (2.78%)	2.00 (0.00)	2.00 (0.00)	2.00 (0.00)	2.00 (0.50)
Phenylketonuria	71 (2.22%)	1.00 (1.00)	1.00 (1.00)	1.00 (1.00)	0.00 (0.50)
Duchenne muscular dystrophy	67 (2.10%)	2.00 (0.50)	2.00 (1.00)	2.00 (0.00)	1.00 (1.00)
Lymphangioleiomyomatosis	57 (1.78%)	2.00 (1.00)	2.00 (1.00)	2.00 (0.25)	2.00 (0.00)
Idiopathic hypogonadotropic hypogonadism	49 (1.53%)	2.00 (1.00)	2.00 (0.00)	1.00 (1.00)	2.00 (0.00)
Prader–Willi syndrome	46 (1.44%)	1.00 (1.00)	1.00 (0.75)	1.00 (1.00)	2.00 (2.00)
Congenital adrenal hypoplasia	44 (1.38%)	2.00 (1.00)	2.00 (1.00)	2.00 (1.00)	2.00 (0.00)
Tuberous sclerosis complex	38 (1.19%)	2.00 (0.00)	2.00 (0.75)	2.00 (0.00)	2.00 (0.25)
Spinal muscular atrophy	30 (0.94%)	1.50 (1.00)	2.00 (1.00)	1.00 (1.00)	1.50 (0.50)
Kallmann syndrome	26 (0.81%)	2.00 (1.00)	2.00 (1.00)	2.00 (0.25)	1.50 (1.25)
Homozygous hypercholesterolemia	25 (0.78%)	2.00 (1.00)	2.00 (0.75)	1.00 (1.00)	2.00 (0.50)
Hereditary epidermolysis bullosa	23 (0.72%)	2.00 (1.00)	2.00 (1.00)	1.00 (1.00)	–
Fabry disease	20 (0.63%)	2.00 (0.25)	2.00 (0.00)	2.00 (0.75)	2.00 (1.00)
Huntington disease	17 (0.53%)	2.00 (1.00)	2.00 (1.00)	2.00 (1.00)	1.00 (0.50)
Gaucher disease	16 (0.50%)	2.00 (1.25)	2.00 (1.00)	3.00 (0.75)	2.00 (0.00)
Spinocerebellar ataxia	15 (0.47%)	1.00 (1.50)	1.00 (2.00)	0.50 (1.00)	0.50 (0.50)
Niemann-Pick disease	12 (0.38%)	3.00 (1.00)	3.00 (1.00)	3.00 (1.00)	–
Mucopolysaccharidosis	6 (0.19%)	2.00 (0.75)	2.00 (0.00)	2.50 (0.50)	2.50 (0.50)
Severe myoclonic epilepsy in infaricy	6 (0.19%)	2.00 (1.50)	2.00 (0.00)	1.00 (1.00)	2.00 (1.00)
Hyperphenylalaninemia	5 (0.16%)	2.00 (1.00)	1.00 (0.00)	2.50 (0.50)	2.00 (0.00)
Spinal and bulbar muscular atrophy	4 (0.13%)	1.00 (0.25)	0.50 (0.50)	1.00 (0.00)	–
Type II glycogen storage disease	3 (0.09%)	1.00 (0.50)	1.00 (0.00)	1.50 (0.50)	–
Tetrahydrobiopterin deficiency	1 (0.03%)	1.00 (0.00)	1.00 (0.00)	–	–

–No physicians answered the relevant knowledge questions

Table 3 compares the characteristics of physicians with a total knowledge score ranging from 0 to 3 as a categorical variable. In this study, 1261 (39.44%) of the involved physicians obtained a total knowledge score of 2, followed by 858 (26.84%) with a score of 1, 854 (26.71%) with a score of 3, and 224 (7.01%) with a score of 0. Significant differences between different groups of physicians were found in gender, birthplace, monthly income, province, education, hospital class, and specialty. Factors associated with the total knowledge score among physicians were identified in Table 4. The variables that were not significantly associated with the total knowledge score in the univariable analysis were not included in the multiple linear regression analysis. In the multiple linear regression analysis, females were found to acquire a higher knowledge score than males (β = 0.08, *p* < 0.05). Compared to physicians with a monthly income of below 5000 RMB, those with an income of 5000–10,000 RMB (β = 0.11, *p* < 0.01) and 10,000–30,000 RMB (β = 0.14, *p* < 0.05) were at a higher probability of obtaining a higher score. Physicians who have worked longer than 30 years were less likely to receive a higher knowledge score than those with working years of 5 or below (β = − 0.18, *p* < 0.05). In contrast to the specialty of internal medicine, specialties of physicians who received a relatively higher score included obstetrics and gynecology (β = − 0.15, *p* < 0.05), radiology (β = − 0.16, *p* < 0.05), intensive care unit (β = − 0.20, *p* < 0.05) and surgery (β = − 0.24, *p* < 0.001). All the variables in the reported model were found to have a value of VIF below 5, which indicated no problem of collinearity.

**Table 3:** Characteristics of physicians with a total knowledge score ranging from a score of 0 to 3

Characteristics	Score of 0	Score of 1	Score of 2	Score of 3	*p*-value
	*Number (percent)*				
*Physician number*	224 (7.01%)	858 (26.84%)	1261 (39.44%)	854 (26.71%)	
*Gender*					< 0.001
Male	118 (8.10%)	417 (28.64%)	580 (39.84%)	341 (23.42%)	
Female	106 (6.09%)	441 (25.33%)	681 (39.12%)	513 (29.47%)	
*Age*					0.254
18–25	5 (12.20%)	10 (24.39%)	15 (36.59%)	11 (26.83%)	
26–35	93 (6.53%)	376 (26.40%)	554 (38.90%)	401 (28.16%)	
36–45	64 (5.95%)	302 (28.09%)	434 (40.37%)	275 (25.58%)	
46–55	47 (8.64%)	145 (26.65%)	214 (39.34%)	138 (25.37%)	
56–65	14 (13.21%)	24 (22.64%)	42 (39.62%)	26 (24.53%)	
66 +	1 (14.29%)	1 (14.29%)	2 (28.57%)	3 (42.86%)	
*Birthplace*					0.045
Urban area	192 (6.75%)	774 (27.22%)	1133 (39.85%)	744 (26.17%)	
Rural area	31 (8.83%)	84 (23.93%)	127 (36.18%)	109 (31.05%)	
Others	1 (33.33%)	0 (0.00%)	1 (33.33%)	1 (33.33%)	
*Monthly income*					< 0.001
< 5000	70 (10.72%)	187 (28.64%)	241 (36.91%)	155 (23.74%)	
5000–10,000	133 (6.56%)	538 (26.55%)	783 (38.65%)	572 (28.23%)	
10,000–30,000	19 (3.75%)	131 (25.89%)	233 (46.05%)	123 (24.31%)	
30,000–50,000	2 (28.57%)	0 (0.00%)	2 (28.57%)	3 (42.86%)	
> 50,000	0 (0.00%)	2 (40.00%)	2 (40.00%)	1 (20.00%)	
*Province*					< 0.001
Jiangsu	56 (4.69%)	303 (25.36%)	498 (41.67%)	338 (28.28%)	
Jiangxi	39 (8.33%)	122 (26.07%)	172 (36.75%)	135 (28.85%)	
Shandong	20 (6.71%)	99 (33.22%)	101 (33.89%)	78 (26.17%)	
Shanxi	95 (9.91%)	259 (27.01%)	371 (38.69%)	234 (24.40%)	
Shannxi	7 (7.07%)	29 (29.29%)	40 (40.40%)	23 (23.23%)	
Sichuan	7 (3.93%)	46 (25.84%)	79 (44.38%)	46 (25.84%)	
*Education*					< 0.001
Secondary vocational school	2 (66.67%)	1 (33.33%)	0 (0.00%)	0 (0.00%)	
Three-year college	2 (10.53%)	8 (42.11%)	7 (36.84%)	2 (10.53%)	
Bachelor’s degree	111 (10.05%)	327 (29.62%)	389 (35.24%)	277 (25.09%)	
Master’s degree	94 (5.27%)	449 (25.20%)	741 (41.58%)	498 (27.95%)	
Doctorate/postdoc	15 (5.19%)	73 (25.26%)	124 (42.91%)	77 (26.64%)	
*Title*					0.153
Primary	69 (8.38%)	213 (25.88%)	302 (36.70%)	239 (29.04%)	
Middle	72 (5.96%)	338 (27.98%)	500 (41.39%)	298 (24.67%)	
Vice-senior	47 (6.63%)	194 (27.36%)	285 (40.20%)	183 (25.81%)	
Senior	36 (8.28%)	108 (24.83%)	163 (37.47%)	128 (29.43%)	
None	0 (0.00%)	5 (22.73%)	11 (50.00%)	6 (27.27%)	
*Working years*					0.348
≤ 5	59 (7.13%)	203 (24.52%)	323 (39.01%)	243 (29.35%)	
6–10	41 (5.26%)	222 (28.50%)	316 (40.56%)	200 (25.67%)	
11–15	36 (7.00%)	146 (28.40%)	200 (38.91%)	132 (25.68%)	
16–20	26 (7.07%)	106 (28.80%)	144 (39.13%)	92 (25.00%)	
21–25	26 (7.81%)	87 (26.13%)	122 (36.64%)	98 (29.43%)	
26–30	17 (7.87%)	55 (25.46%)	91 (42.13%)	53 (24.54%)	
30 +	19 (11.95%)	39 (24.53%)	65 (40.88%)	36 (22.64%)	
*Hospital class*					0.008
Primary hospital	2 (50.00%)	1 (25.00%)	1 (25.00%)	0 (0.00%)	
Secondary hospital	2 (25.00%)	4 (50.00%)	1 (12.50%)	1 (12.50%)	
Tertiary hospital	220 (6.91%)	853 (26.78%)	1259 (39.53%)	853 (26.78%)	
*Specialty*					< 0.001
Internal medicine	59 (4.31%)	325 (23.72%)	563 (41.09%)	423 (30.88%)	
Anesthesiology	3 (9.09%)	15 (45.45%)	12 (36.36%)	3 (9.09%)	
Dermatology	0 (0.00%)	7 (12.28%)	31 (54.39%)	19 (33.33%)	
Emergency medicine	6 (6.74%)	23 (25.84%)	40 (44.94%)	20 (22.47%)	
Infectious dept	5 (7.25%)	9 (13.04%)	34 (49.28%)	21 (30.43%)	
Intensive care unit	7 (8.14%)	29 (33.72%)	30 (34.88%)	20 (23.26%)	
Laboratory dept	0 (0.00%)	1 (33.33%)	2 (66.67%)	0 (0.00%)	
Obstetrics and gynecology	13 (9.77%)	40 (30.08%)	50 (37.59%)	30 (22.56%)	
Oncology	6 (8.11%)	29 (39.19%)	20 (27.03%)	19 (25.68%)	
Ophthalmology	2 (2.90%)	19 (27.54%)	28 (40.58%)	20 (28.99%)	
Orthopedic surgery, medical cosmetology	3 (27.27%)	1 (9.09%)	3 (27.27%)	4 (36.36%)	
Otolaryngology	6 (16.22%)	11 (29.73%)	12 (32.43%)	8 (21.62%)	
Pain medicine	0 (0.00%)	0 (0.00%)	5 (83.33%)	1 (16.67%)	
Pathology	0 (0.00%)	1 (12.50%)	7 (87.50%)	0 (0.00%)	
Pediatrics	26 (8.02%)	69 (21.30%)	119 (36.73%)	110 (33.95%)	
Psychiatry	2 (11.76%)	8 (47.06%)	4 (23.53%)	3 (17.65%)	
Radiology	13 (9.42%)	46 (33.33%)	59 (42.75%)	20 (14.49%)	
Sports medicine, rehabilitation	12 (13.95%)	29 (33.72%)	32 (37.21%)	13 (15.12%)	
Stomatology	5 (17.24%)	8 (27.59%)	12 (41.38%)	4 (13.79%)	
Surgery	41 (9.88%)	145 (34.94%)	149 (35.90%)	80 (19.28%)	
Traditional Chinese medicine	5 (12.82%)	16 (41.03%)	12 (30.77%)	6 (15.38%)	
Others	10 (9.62%)	27 (25.96%)	37 (35.58%)	30 (28.85%)

**Table 4:** Factors associated with the total knowledge score among physicians in China

Variables	Coefficients [95%CI]	
	Univariable	Multivariable
*Gender*		
Male	Reference	Reference
Female	0.13 [0.07, 0.20]***	0.08 [0.01, 0.14]*
*Age*		
18–25	Reference	
26–35	0.11 [− 0.17, 0.38]	
36–45	0.08 [− 0.20, 0.35]	
46–55	0.03 [− 0.25, 0.32]	
56–65	− 0.03 [− 0.35, 0.30]	
66 +	0.22 [− 0.50, 0.93]	−
*Birthplace*		
Urban area	Reference	−
Rural area	0.04 [− 0.06, 0.14]	
Others	− 0.19 [− 1.20, 0.82]	
*Monthly income*		
< 5000	Reference	Reference
5000–10,000	0.15 [0.07, 0.23]***	0.11 [0.03, 0.18]**
10,000–30,000	0.17 [0.07, 0.28]**	0.14 [0.03, 0.25]*
30,000–50,000	0.12 [− 0.54, 0.78]	− 0.03 [− 0.62, 0.56]
> 50,000	0.06 [− 0.72, 0.85]	0.21 [− 0.48, 0.91]
*Province*		
Jiangsu	Reference	Reference
Shandong	− 0.14 [− 0.25, − 0.03]*	− 0.10 [− 0.20, 0.00]
Jiangxi	− 0.07 [− 0.17, 0.02]	− 0.04 [− 0.13, 0.05]
Shanxi	− 0.16 [− 0.24, − 0.08]***	− 0.10 [− 0.18, − 0.03]**
Sichuan	− 0.01 [− 0.15, 0.13]	0.02 [− 0.11, 0.15]
Shannxi	− 0.14 [− 0.32, 0.04]	− 0.19 [− 0.36, − 0.01]*
*Education*		
Secondary vocational school	Reference	Reference
Three-year college	1.14 [0.06, 2.22]*	0.36 [− 0.64, 1.37]
Bachelor	1.42 [0.41, 2.43]**	0.53 [− 0.41, 1.47]
Master	1.59 [0.58, 2.59]**	0.63 [− 0.31, 1.57]
Doctor/postdoc	1.58 [0.57, 2.59]**	0.61 [− 0.33, 1.56]
*Title*		
Primary	Reference	−
Middle	− 0.02 [− 0.10, 0.06]	
Vice-senior	− 0.01 [− 0.10, 0.08]	
Senior	0.02 [− 0.09, 0.12]	
None	0.18 [− 0.20, 0.56]	
*Working years*		
≤ 5	Reference	Reference
6–10	− 0.04 [− 0.13, 0.05]	− 0.05 [− 0.13, 0.03]
11–15	− 0.07 [− 0.17, 0.03]	0.00 [− 0.10, 0.09]
16–20	− 0.09 [− 0.19, 0.02]	− 0.05 [− 0.16, 0.05]
21–25	− 0.03 [− 0.14, 0.08]	0.01 [− 0.09, 0.12]
26–30	− 0.07 [− 0.21, 0.06]	− 0.04 [− 0.17, 0.09]
30 +	− 0.16 [− 0.32, − 0.01]*	− 0.18 [− 0.32, − 0.03]*
*Hospital class*		
Primary hospital	Reference	Reference
Secondary hospital	0.38 [− 0.69, 1.44]	0.19 [− 0.79, 1.17]
Tertiary hospital	1.11 [0.24, 1.99]*	0.33 [− 0.48, 1.15]
*Specialty*		
Internal medicine	Reference	Reference
Anesthesiology	− 0.53 [− 0.83, − 0.23]**	− 0.45 [− 0.72, − 0.17]**
Dermatology	0.23 [− 0.01, 0.46]	0.18 [− 0.04, 0.40]
Emergency medicine	− 0.15 [− 0.34, 0.03]	− 0.11 [− 0.29, 0.06]
Infectious disease	0.04 [− 0.17, 0.26]	− 0.06 [− 0.26, 0.14]
Intensive care unit	− 0.25 [− 0.44, − 0.06]*	− 0.20 [− 0.37, − 0.02]*
Laboratory department	− 0.32 [− 1.31, 0.68]	− 0.13 [− 1.03, 0.77]
Obstetrics and gynecology	− 0.26 [− 0.41, − 0.10]**	− 0.15 [− 0.30, − 0.01]*
Oncology	− 0.28 [− 0.49, − 0.08]**	− 0.38 [− 0.56, − 0.19]***
Ophthalmology	− 0.03 [− 0.24, 0.18]	0.00 [− 0.20, 0.20]
Orthopedic surgery, medical cosmetology	− 0.26 [− 0.78, 0.26]	− 0.20 [− 0.66, 0.27]
Otolaryngology	− 0.39 [− 0.68, − 0.10]**	− 0.46 [− 0.72, − 0.20]**
Pain medicine	0.18 [− 0.52, 0.89]	0.36 [− 0.27, 0.99]
Pathology	− 0.11 [− 0.72, 0.50]	− 0.44 [− 1.00, 0.11]
Pediatrics	− 0.02 [− 0.13, 0.09]	0.08 [− 0.02, 0.19]
Psychiatry	− 0.51 [− 0.93, − 0.09]*	− 0.43 [− 0.81, − 0.05]*
Radiology	− 0.36 [− 0.52, − 0.21]***	− 0.16 [− 0.30, − 0.02]*
Sports medicine, rehabilitation	− 0.45 [− 0.64, − 0.26]***	− 0.16 [− 0.33, 0.02]
Stomatology	− 0.47 [− 0.79, − 0.15]**	− 0.63 [− 0.93, − 0.34]***
Surgery	− 0.34 [− 0.44, − 0.24]***	− 0.24 [− 0.34, − 0.14]***
Traditional Chinese medicine	− 0.50 [− 0.78, − 0.22]***	− 0.19 [− 0.45, 0.06]
Others	− 0.15 [− 0.32, 0.03]	− 0.06 [− 0.22, 0.10]
*Rare disease type*		
Albinism	Reference	Reference
Osteogenesis imperfecta	− 0.29 [− 0.45, − 0.13]***	− 0.28 [− 0.44, − 0.12]**
Homozygous hypercholesterolemia	− 0.27 [− 0.59, 0.05]	− 0.42 [− 0.74, − 0.10]*
Duchenne muscular dystrophy	0.11 [− 0.10, 0.31]	− 0.09 [− 0.30, 0.12]
Multiple sclerosis	− 0.53 [− 0.64, − 0.42]***	− 0.64 [− 0.75, − 0.52]***
Fabry disease	0.26 [− 0.09, 0.62]	0.07 [− 0.28, 0.42]
Hepatolenticular degeneration	0.38 [0.27, 0.48]***	0.25 [0.13, 0.36]***
Gaucher disease	0.15 [− 0.25, 0.55]	− 0.04 [− 0.43, 0.36]
Huntington disease	− 0.02 [− 0.41, 0.36]	− 0.03 [− 0.41, 0.35]
Amyotrophic lateral sclerosis	− 0.22 [− 0.38, − 0.07]**	− 0.33 [− 0.49, − 0.17]***
Spinocerebellar ataxia	− 0.65 [− 1.06, − 0.24]**	− 0.69 [− 1.10, − 0.29]**
Spinal muscular atrophy	− 0.39 [− 0.68, − 0.09]*	− 0.50 [− 0.80, − 0.21]**
Spinal and bulbar muscular atrophy	− 1.04 [− 1.82, − 0.25]**	− 1.00 [− 1.78, − 0.23]*
Tuberous sclerosis complex	0.03 [− 0.24, 0.29]	− 0.10 [− 0.37, 0.16]
Kallmann syndrome	0.02 [− 0.29, 0.34]	− 0.14 [− 0.46, 0.17]
Langerhans cell histiocytosis	0.60 [0.44, 0.77]***	0.55 [0.38, 0.72]***
Lymphangioleiomyomatosis	− 0.12 [− 0.34, 0.10]	− 0.28 [− 0.50, − 0.06]*
Marfan syndrome	0.07 [− 0.06, 0.19]	0.00 [− 0.13, 0.12]
Niemann-Pick disease	0.71 [0.26, 1.17]**	0.64 [0.18, 1.09]**
Mucopolysaccharidosis	0.55 [− 0.10, 1.19]	0.31 [− 0.32, 0.95]
Prader–Willi syndrome	− 0.53 [− 0.77, − 0.29]***	− 0.79 [− 1.04, − 0.55]***
General myathenic gravis	0.08 [− 0.07, 0.22]	0.05 [− 0.10, 0.20]
Neuromyelitis optica	0.20 [0.02, 0.38]*	0.03 [− 0.16, 0.21]
Type II glycogen storage disease	− 0.45 [− 1.36, 0.45]	− 0.66 [− 1.55, 0.23]
Idiopathic hypogonadotropic hypogonadism	− 0.09 [− 0.33, 0.14]	− 0.28 [− 0.52, − 0.05]*
Idiopathic pulmonary arterial hypertension	0.48 [0.34, 0.62]***	0.33 [0.19, 0.48]***
Systemic sclerosis	0.50 [0.36, 0.65]***	0.32 [0.17, 0.47]***
Congenital adrenal hypoplasia	0.24 [− 0.01, 0.48]	0.01 [− 0.23, 0.26]
Hemophilia	0.64 [0.52, 0.76]***	0.58 [0.46, 0.69]***
Hereditary epidermolysis bullosa	− 0.35 [− 0.69, − 0.02]*	− 0.52 [− 0.85, − 0.19]**
Severe myoclonic epilepsy in infaricy	− 0.45 [− 1.10, 0.19]	− 0.54 [− 1.18, 0.09]
Hyperphenylalaninemia	0.01 [− 0.69, 0.72]	− 0.19 [− 0.88, 0.50]
Phenylketonuria	− 1.13 [− 1.32, − 0.93]***	− 1.18 [− 1.38, − 0.97]***
Tetrahydrobiopterin deficiency	− 0.79 [− 2.35, 0.78]	− 1.05 [− 2.59, 0.48]

*p < 0.05; **p < 0.01; ***p < 0.001

^–^Not included in the multiple linear regression analysis

## Discussion

This study assessed the level of knowledge on rare diseases among Chinese physicians who had medical encounters of 34 rare diseases of concern. Approximately 73% of the invited participants were eligible for answering knowledge questions provided by 34 patient organizations with a specific disease of interest. This may indicate that most physicians will encounter the diagnosis or treatment of a rare disease patient in their professional careers. Among the 3197 eligible participants in this study, over 60% of them obtained a total knowledge score of 2 or 3, which represented an average or good level of rare disease knowledge. Physicians’ knowledge on rare diseases were generally investigated through self-reported questionnaires in previous studies [4, 15, 17]. A study conducted in Belgium revealed that most of the investigated specialists scored their knowledge on rare diseases as average or good [17], while another international study indicated that the majority of specialists rated their current knowledge as excellent or good [4]. These prior studies focused on physicians’ individual assessment of their knowledge on rare diseases in general and the responses can be varied depending on specialty, healthcare system, and the disease in physicians’ mind when filling out the questionnaire [17]. Furthermore, previous studies developed questions to investigate physicians’ education and information needs on rare diseases through expert interviews or previous publications [11, 17], however, these questionnaires may lack patient perspectives. To resolve the above uncertainties, the present study invited patient organizations with a unique disease of interest to develop questions so as to assess levels of disease-specific knowledge among physicians from an objective perspective. The findings in the current study can not only specify physicians’ needs on rare disease knowledge precisely, but also provide insights into the improvement of quality of care for rare disease patients.

Among the 34 rare diseases of concern, the median knowledge score of 10 rare diseases were relatively low. Several factors may account for the low knowledge score of these rare diseases. Firstly, although the prevalence of rare diseases is generally low, the degree of rarity between different diseases still varies significantly, ranging from somewhat rare, rare, to extremely rare [6]. Due to the lack of medical encounters of extremely rare diseases, physicians may have insufficient knowledge on these diseases such as Tetrahydrobiopterin deficiency with a prevalence rate of 1–9/1000,000 [18]. Secondly, technological advances on the diagnosis and treatment of rare diseases are rapidly evolving in recent years, however, physicians may not fully understand the translation process of the new technology into clinical practice, resulting in low correct rates of relevant questions, for instance, Amyotrophic lateral sclerosis Q2 and Multiple sclerosis Q2 (Additional file 2). Third, the majority of rare diseases are genetic in origin, many of which are caused by defects in a single gene or multiple genetic mutations [19]. However, physicians may receive lack of training and knowledge on genetics, resulting in a bad performance in answering inheritance-related questions such as Spinal muscular atrophy Q3, Prader–Willi syndrome Q3, and Spinal and bulbar muscular atrophy Q3 (Additional file 2). Fourth, due to lack of effective treatment for some rare diseases, patients with such diseases can only receive supportive treatment options including nutrition therapies, psychological treatment, and rehabilitation support. This situation often requires multidisciplinary expertise and communication, while insufficient multidisciplinary collaboration may lead to specialists’ limited knowledge on specific rare diseases [11, 15, 17], for example, Phenylketouria Q2 and Q3 (Additional file 2). In general, apart from identifying physicians’ characteristics associated with the knowledge score on rare diseases, it’s also important to investigate the relationship between physicians’ knowledge on rare diseases and disease attributes (e.g., a disease with no treatment or with therapies in development) in future studies.

Factors associated with the knowledge score were identified in the multiple linear regression analysis. Females were more likely to obtain a higher knowledge score than males. Physicians with a monthly income of 5000–10,000 RMB and 10,000–30,000 RMB were associated with a higher knowledge score. This finding suggested that the socioeconomic status of physicians could affect their performance on rare disease knowledge. Hospitals locates in eastern China usually have access to more healthcare resources and insurance subsidies from the government. Hence, physicians from eastern China are more likely to receive a relatively higher income. Due to the uneven distribution of healthcare in China, patients have to travel across regions to access high-quality healthcare for the diagnosis and treatment of rare diseases [16]. Physicians from eastern China have more opportunities to encounter rare disease patients and thus increase related knowledge. On the contrary to our intuition, physicians working 5 years or below were more likely to obtain a higher knowledge score than physicians working longer than 30 years. This finding may indicate that working experience is not enough for accumulating rare disease knowledge. Medical school education and continuing training also play an important role to raise awareness of physicians on rare diseases; such education and training have gradually increased over time [15]. Several specialties of physicians including internal medicine, obstetrics and gynecology, radiology, intensive care unit, and surgery, had a comparably better performance in knowledge score after controlling for the other variables. These specialties of physicians had medical encounters of either a large number of rare disease patients or a great variety of rare diseases, which contributed to the accumulation of rare disease knowledge (Additional file 3).

Although rare diseases only affect a small proportion of the general population, some specialties of physicians may encounter a large number of rare disease patients or a great variety of rare diseases in China. These specialties of physicians may have better performance on specific rare diseases, however, it’s unrealistic for them to be experts for all the rare diseases that they encounter during their professional lives. This situation is not unique to China, but also common in different healthcare systems around the world [20]. Under this scenario, physicians should support the patient participation in medical encounters by sharing knowledge openly and encouraging patients’ self-reliance. Additionally, patient organizations can play a critical role in providing rare disease patients with services and resources that are not otherwise available through expert patients or invited physicians [15, 21, 22]. Apart from the positive sustainment of patients’ information-seeking process, it is equally important to enhance the pooling of knowledge on rare diseases by the implementation of multidisciplinary and digital platform, and to establish an effective referral method at the healthcare system level. Due to the low prevalence and scattered population of rare disease patients in China, tertiary hospitals located in big cities could establish a regular board of advisors by including experts from various specialties to further improve the diagnosis and treatment of rare diseases. An ideal digital platform was described as submitting patients’ symptoms and test results and obtaining a differential diagnosis of rare diseases as output [15, 17]. In China, despite the guideline for the diagnosis and treatment of rare diseases issued in 2019, more detailed information on differential diagnosis of rare diseases, referral services provided by experts or hospitals, and patient organizations are needed to guide medical services for rare diseases. Furthermore, Zhou and Nunes revealed that the key barrier to knowledge sharing in the provision of healthcare referral services in China is lack of leadership of the governance agencies in the process of inter-institutional knowledge sharing at the national and regional levels [23]. These problems need to be addressed to further enhance the implementation of knowledge sharing in the field of rare diseases in China.

To the best knowledge of us, this is the first national study investigating physicians’ knowledge on 34 specific rare diseases in China. The study sample were recruited across eastern, central, and western China, which has increased the representativeness of the research findings. However, there are several limitations to this study. First, the knowledge questions developed in this study have not been validated, resulting in discrepancies in the difficulties of knowledge questions between different rare diseases. Future studies are needed to validate the difficulties of disease-specific knowledge questions so as to make the performance of physicians more comparable. Second, only 3 knowledge questions were used to measure the level of knowledge on a specific rare disease among physicians, lowering the reliability of the study findings. A more comprehensive questionnaire is required to assess the level of physicians’ knowledge on specific rare diseases in future studies. Additionally, physicians were required to select only one rare disease that they felt most familiar with so as to compare their performance on disease-specific knowledge. However, physicians may encounter various types of rare diseases during their professional careers. Apart from their most familiar rare disease, future studies need to investigate physicians’ knowledge on other specific rare diseases that they have encountered during their professional lives. Third, due to the selection bias of the sampling method, most of the participants in this study were specialists from tertiary hospitals in China. Several studies have indicated that the general practitioners’ self-reported knowledge on rare diseases was worse than that of specialists [11, 17]. Hence, the findings reported in the current study are not generalizable to a broader physician population in China. Fourth, selection bias may arise since the physicians included in the study reported their experience with rare diseases by themselves. Physicians that either declared having no medical encounters of rare disease patients or chose other rare diseases as their most familiar one beyond the specific 34 rare diseases of interest were not taken into account according to physicians’ responses in this study. Although these physicians were excluded from the current study, it’s still important to assess their knowledge on specific rare diseases using more objective data in future studies.

## Conclusions

Almost two thirds of the participants had an average or good level of knowledge on the specific rare disease that they felt most experienced in. Physicians with female gender, a monthly income of 5000–10,000 RMB and 10,000–30,000 RMB, and specialties of internal medicine, obstetrics and gynecology, radiology, intensive care unit, and surgery, were associated with a relatively higher knowledge score. Medical education and continuous training programs, especially on genetics and translational research, are further needed to increase physicians’ knowledge on rare diseases. Also, there are urgent needs of high-quality clinical guidelines and multidisciplinary collaboration to provide guidance on medical services for rare diseases in the healthcare system of China.

## Supplementary Information

**Additional file 1.** List of knowledge questions and answers based on 34 rare diseases of concern.

**Additional file 2.** Correct rates of answering each of the three knowledge questions among physicians based on rare diseases.

**Additional file 3.** Frequencies of physicians in different specialties based on 34 rare diseases of concern.

## Data Availability

All data generated or analyzed during this study are included in this published article and its Additional files.
