# Supplementary material for: Physicians’ knowledge on specific rare diseases and its associated factors: a national cross-sectional study from China
Source: Orphanet J Rare Dis. 2022 Mar 5;17:120. doi: 10.1186/s13023-022-02243-7 (PMC8898513; doi:10.1186/s13023-022-02243-7)
Supplement: Supplementary file 1 — Additional file 1. List of knowledge questions and answers based on 34 rare diseases of concern. [file 13023_2022_2243_MOESM1_ESM.docx]

**Additional file 1. List of knowledge questions and answers based on 34 rare diseases of concern**

| **罕见病种类**  **（Type of rare diseases）** | **知识题** | **Knowledge questions** | **正确答案（Answers）** |
| --- | --- | --- | --- |
| 白化病（Albinism） | 1. 白化病只传男不传女 | 1. Albinism affects males only who have inherited an albinism gene from their mothers. | FALSE |
|  | 2. 白化病解决了眼球震颤的问题之后，视力就正常了 | 2. The visual impairment will return to normal if the nystagmus problem is resolved in patients with Albinism. | FALSE |
|  | 3. 白化病都是由同一个基因缺陷导致的 | 3. Albinism is caused by the same gene defect. | FALSE |
| 成骨不全症（Osteogenesis Imperfecta, OI） | 1. 成骨不全症有有效的治疗方法 | 1. There are effective treatments to cure osteogenesis imperfecta (OI). | FALSE |
|  | 2. 瓷娃娃手术治疗不能使用钢板手术 | 2. Patients with OI, namely porcelain doll, should not use steel plates in the surgery. | TRUE |
|  | 3. 磷酸类药物是成骨不全症的特效药 | 3. Phosphoric acid drugs are specific medicine for OI. | FALSE |
| 纯合子家族性高胆固醇血症（Homozygous Hypercholesterolemia） | 1. 纯合子家族性高胆固醇血症是一种遗传疾病 | 1. Homozygous hypercholesterolemia is a genetic disorder. | TRUE |
|  | 2. 纯合子家族性高胆固醇血症仅在有皮肤黄色瘤表现时严重危害心血管健康 | 2. Homozygous hypercholesterolemia is a serious cardiovascular hazard only in the presence of xanthoma in the skin. | FALSE |
|  | 3. 荷兰脂质网络评分标准大于5分即可确诊为家族性高胆固醇血症 | 3. Homozygous hypercholesterolemia is diagnosed with the Dutch Lipid Network score more than 5 points. | FALSE |
| 杜氏肌营养不良（Duchenne Muscular Dystrophy, DMD） | 1. 杜氏肌营养不良都是由于遗传所致的 | 1. Duchenne muscular dystrophy (DMD) is all caused by genetic factors. | FALSE |
|  | 2. 杜氏肌营养不良的患者，其肌酸激酶越低越好 | 2. Of patients with DMD, the lower the creatine kinase levels, the better. | FALSE |
|  | 3. 杜氏肌营养不良基因突变类型有缺失、重复和点突变三种。无义突变是点突变中的一种 | 3. There are three types of gene mutations in DMD, namely, deletion, duplication, and point mutation. Nonsense mutation is one kind of the point mutations. | TRUE |
| 多发性硬化（Multiple Sclerosis, MS） | 1. 多发性硬化是慢性终身性疾病 | 1. Multiple sclerosis (MS) is a chronic and lifelong disease. | TRUE |
|  | 2. 多发性硬化有特定的MS生物标志物 | 2. MS has specific biomarkers | FALSE |
|  | 3. 多发性硬化的脱髓鞘主要由少突胶质细胞损伤引起 | 3. Demyelination in MS is mainly induced by oligodendrocyte injury. | FALSE |
| 法布雷病（Fabry Disease） | 1. 法布雷病可以通过肢端疼痛红肿、少汗无汗等临床表现来确诊 | 1. Fabry disease can be diagnosed by the clinical manifestations of redness, pain, swelling in the extremities, and decreased or absent sweat production. | FALSE |
|  | 2. 已经有特效药能够治愈法布雷病 | 2. Specific medicine is available to cure Fabry disease. | FALSE |
|  | 3. 作为一种罕见的X染色体连锁遗传的鞘糖脂类代谢疾病，某男性基因检测GLA有突变，但酶活性正常，他应该被确诊为法布雷患者 | 3. Fabry disease is a rare X-chromosomal-linked inherited disorder of glycosphingolipid metabolism. If a male has the α-galactosidase A (GLA) gene mutation but normal α-GLA enzymatic activity, he should be diagnosed with Fabry disease. | FALSE |
| 肝豆状核变性（Hepatolenticular Degeneration） | 1. 肝豆状核变性是常染色体隐性遗传病。 | 1. Hepatolenticular degeneration is an autosomal recessive genetic disease | TRUE |
|  | 2. 所有肝豆状核变性的患者都有角膜 K-F 环这一临床表现 | 2. All the patients with Hepatolenticular degeneration have a clinical feature of Kayser-Fleischer rings of cornea (KF rings). | FALSE |
|  | 3. 肝移植是治疗肝豆状核变性的最主要的手段 | 3.Liver transplantation is the main treatment option for patients with Hepatolenticular degeneration. | FALSE |
| 戈谢病（Gaucher Disease） | 1. 戈谢病是常染色体隐性遗传病。 | 1. Gaucher disease is an autosomal recessive inherited disease. | TRUE |
|  | 2. 戈谢病患者都是从儿童时开始发病的 | 2. All the patients with Gaucher disease start to develop symptoms from childhood. | FALSE |
|  | 3. 酶替代治疗对所有戈谢病类型的患者都适用 | 3. Enzyme replacement therapy is appropriate for all types of Gaucher disease. | FALSE |
| 亨廷顿舞蹈病（Huntington Disease） | 1. 亨廷顿舞蹈症是家族遗传病。 | 1.Huntington Disease is a familial-hereditary disease. | TRUE |
|  | 2. 基因检测CAG重复数大于40的患者不一定会发病 | 2.Patients with more than 40 CAG repeats detected through genetic testing may not develop the signs and symptoms of Huntington disease. | FALSE |
|  | 3. 亨廷顿舞蹈症的症状是由于基因里的CAG过度重复，导致大脑里大量有毒蛋白堆积 | 3. The symptoms of Huntington disease are caused by an over-duplication of the CAG gene, which causes the accumulation of toxic protein deposits. | TRUE |
| 肌萎缩侧索硬化（Amyotrophic Lateral Sclerosis, ALS） | 1. ALS特指运动神经元病。 | 1. Amyotrophic lateral sclerosis (ALS) refer to motor neuron disease in particular | FALSE |
|  | 2. 基因检测是ALS的最主要的确诊手段。 | 2. Genetic testing is the main diagnostic method for ALS. | FALSE |
|  | 3. 目前而言，心理支持、营养支持、呼吸支持、康复护理支持，是延长渐冻人生存时间，提高生存质量的科学有效途径。 | 3. At present, psychological support, nutritional support, respiratory support and rehabilitation support are scientific and effective ways to prolong the survival time and improve the quality of life of ALS patients. | TRUE |
| 脊髓小脑性共济失调（Spinocerebellar Ataxia, SCA） | 1. 脊髓小脑性共计失调是一种常染色体隐性遗传病。 | 1. Spinocerebellar ataxia (SCA) is an autosomal recessive genetic disorder. | FALSE |
|  | 2. 脊髓小脑性共计失调属于有药可医的罕见病。 | 2. SCA is a rare disease that can be treated with drugs. | FALSE |
|  | 3. 在我国，最常见的脊髓小脑性共济失调的亚型是SCA1型。 | 3. In China, the most common subtype of SCA is SCA1. | FALSE |
| 脊髓性肌萎缩症（Spinal Muscular Atrophy, SMA） | 1. 只有男孩子才会得脊髓性肌萎缩症 | 1. Only boys can be diagnosed with spinal muscular atrophy (SMA). | FALSE |
|  | 2. 脊髓性肌萎缩症是没有办法治疗的 | 2. There is no cure for patients with SMA. | FALSE |
|  | 3. 脊髓性肌萎缩症是常染色体隐性遗传，主要由SMN基因第7号外显子纯合缺失引起 | 3. SMA is an autosomal recessive inheritance caused by homozygous deletion of exon 7 of the SMN gene. | FALSE |
| 脊髓延髓肌萎缩症（Spinal and Bulbar Muscular Atrophy, Kennedy’s disease） | 1. 肯尼迪病是因为美国总统约翰•肯尼迪患有该疾病而得名 | 1. Kennedy's disease is named after U.S. President John F. Kennedy, who suffered from the disease. | FALSE |
|  | 2. 肯尼迪病患者的CAG重复数越大，则病情发展越快 | 2. The greater the number of CAG repeats in patients with Kennedy's disease, the faster the disease progresses. | FALSE |
|  | 3. 根据遗传规律可以得知，肯尼迪患者的外孙的患病概率为50%，外孙女为携带者的概率为50% | 3. According to the rules of heredity, a grandson of a patient with Kennedy's disease has a 50% chance of developing the disease and a 50% chance of a granddaughter being a carrier. | TRUE |
| 结节性硬化症（Tuberous Sclerosis Complex, TSC） | 1. 结节性硬化症是一种常染色体显性遗传性疾病,以多器官系统的错构瘤为特征,主要包括大脑、皮肤、心脏、肾脏和肺等部位 | 1. Tuberous sclerosis (TSC) is an autosomal dominant genetic disorder characterized by hamartomas in multiple organ systems, mainly including the brain, skin, heart, kidney, and lung. | TRUE |
|  | 2. 常见的引起结节性硬化症的基因突变位点有TSC1和TSC2，主要致病基因位于9和13号染色体 | 2. Common genetic mutations that cause TSC are TSC1 and TSC2, and the main pathogenic genes located on chromosome 9 and 13. | FALSE |
|  | 3. 结节性硬化症患者会出现智力低下、癫痫、自闭症等临床症状 | 3. Patients with TSC suffer from mental retardation, epilepsy, autism, and other clinical symptoms. | TRUE |
| 卡尔曼综合征（Kallmann Syndrome） | 1. 卡尔曼氏综合征染色体数量是正常的。 | 1. The number of chromosomes in patients with Kallman syndrome is normal. | TRUE |
|  | 2. 卡尔曼氏综合征的嗅觉经过治疗可以完全恢复。 | 2. The impaired sense of smell in patients with Kallmann syndrome can be completely cured by treatment. | FALSE |
|  | 3. 促性腺激素替代治疗对所有卡尔曼氏综合征患者都适用。 | 3. Gonadotropin replacement therapy is appropriate to all patients with Kallman syndrome | TRUE |
| 朗格汉斯组织细胞增生症（Langerhans Cell Histiocytosis） | 1. 确诊朗格汉斯组织细胞增生症的金标准是病理活检 | 1. Pathological diagnosis is the golden diagnosis standard for Langerhans cell histiocytosis (LCH). | TRUE |
|  | 2. 朗格汉斯组织细胞增生症患者有骨损症状的，一定要做骨刮除手术和骨修补手术 | 2. Patients with bone LCH lesions must be treated with bone curettage and repair surgery. | FALSE |
|  | 3. 朗格汉斯组织细胞增生症患者BRAF V600E的基因突变较多，此突变基因更容易造成患者受累器官持久，不可逆的损伤，如神经病变，垂体性尿崩症，内脏等多器官受累 | 3. Most of the patients with LSH have mutations in BRAF V600E gene, which is more likely to cause lasting and irreversible damage to the affected organs, such as neuropathy, pituitary diabetes insipidus, internal organs and other organs. | TRUE |
| 淋巴管肌瘤病（Lymphangioleiomyomatosis, LAM） | 1. 淋巴管肌瘤病是一种高发于育龄期女性的低度恶性肿瘤 | 1. Lymphangiomyomatosis (LAM) is a kind of low grade malignant tumor that occurs most frequently in females of childbearing age. | TRUE |
|  | 2. 西罗莫司（雷帕霉素）是目前唯一能延缓疾病进展的药物 | 2. Sirolimus (rapamycin) is the only drug that can slow the progression of LAM currently. | TRUE |
|  | 3. 淋巴管肌瘤病患者中位生存期约为19年 | 3. The median survival year of patients with LAM is about 19 years. | FALSE |
| 马方综合征（Marfan Syndrome） | 1. 如果家族中有人被诊断为马凡氏综合征，应对其父母以及孩子进行排查 | 1. If a person has been diagnosed with Marfan syndrome, their parents and children should be screened. | TRUE |
|  | 2. 当马凡患者主动脉扩张达到4.5CM以上时，要对其进行手术干预 | 2. Surgical interventions should be performed in patients with Marfan syndrome when aortic dilatation reaches more than 4.5cm. | TRUE |
|  | 3. 导致马凡氏综合征的根本原因是微纤维蛋白-2的基因突变 | 3. Mutations in the microfibrin-2 gene is the main cause to induce Marfan syndrome. | FALSE |
| 尼曼匹克病（Niemann-Pick Disease） | 1. 尼曼匹克病是为常染色体隐性遗传病 | 1. Niemann-Pick disease is an autosomal recessive genetic disorder | TRUE |
|  | 2. 尼曼匹克患者经骨髓穿刺检查可找到泡沫细胞 | 2. Foam cells can be detected through bone marrow punctures in patients with Niemann-Pick disease. | TRUE |
|  | 3. 尼曼匹克病B型患者均会出现智力减退、语言障碍、学习困难、步态不稳等累及神经系统方面的症状 | 3. All the patients with Niemann-Pick disease type B will suffer from mental decline, speech problems, learning difficulties, gait instability and other symptoms involving the nervous system. | FALSE |
| 黏多糖贮积症（Mucopolysaccharidosis） | 1. 黏多糖贮积症属于基因突变造成的遗传代谢疾病 | 1.Mucopolysaccharidoses are a group of inherited metabolic diseases caused by gene mutation. | TRUE |
|  | 2. 黏多糖贮积症现在常见的有7种类型 | 2.Seven common subtypes of mucopolysaccharidoses have been identified. | TRUE |
|  | 3. 黏多糖贮积症的诊断只能依靠基因检测 | 3. Diagnosis of a mucopolysaccharidosis disorder is dependent on genetic testing. | FALSE |
| 小胖威利综合征（Prader-Willi Syndrome） | 1. 小胖威利患儿平常摄入的热量是同龄人的60%左右 | 1. Children with Prader-Willi Syndrome typically consume about 60% of the calories of their peers. | TRUE |
|  | 2. 婴儿时期的小胖威利患儿皮肤较为苍白，且有肌肉高张力的现象 | 2. Children with Prader-Willi syndrome have unusually fair skin and excessive muscle tone. | FALSE |
|  | 3. 小胖威利是由于来自父亲的十五号染色体有缺失而造成的 | 3. Prader-Willi syndrome is caused by the missing genetic information from the copy of chromosome 15 inherited from the father. | FALSE |
| 重症肌无力（General Myathenic Gravis） | 1. 重症肌无力是指各种严重的肌肉无力病症 | 1. General myasthenia gravis refers to a variety of severe muscle weakness conditions. | FALSE |
|  | 2. 重症肌无力可以治疗和康复的 | 2. General myasthenia gravis can be treated and recovered | TRUE |
|  | 3. 可以通过肌电图来完全确诊重症肌无力 | 3. General myasthenia gravis can be diagnosed by electromyography. | FALSE |
| 视神经脊髓炎（Neuromyelitis Optica, NMO） | 1. 视神经脊髓炎是自身免疫性疾病 | 1. Neuromyelitis optica (NMO) is an autoimmune disease. | TRUE |
|  | 2. 视神经脊髓炎没有特定的生物标志物 | 2. There is no specific biomarker for NMO. | FALSE |
|  | 3. 视神经脊髓炎的脱髓鞘主要由少突胶质细胞损伤引起 | 3. Demyelination of NMO is mainly caused by oligodendrocyte injury. | FALSE |
| 糖原累积病（Type II Glycogen Storage Disease, Pompe disease） | 1. 庞贝是有药可医的罕见病 | 1.Pompe disease is a rare disease with specific medicine. | TRUE |
|  | 2. 庞贝患者的遗传方式为显性遗传 | 2.Pompe disease is a genetic disease that is inherited in dominant pattern. | FALSE |
|  | 3. 婴儿型庞贝进展较快的原因是酶活性更低 | 3.The reason for the rapid progression of infantile-onset Pompe disease is the lower enzyme activity. | FALSE |
| 特发性低促性腺激素性性腺功能减退症（Idiopathic Hypogonadotropic Hypogonadism, IHH） | 1. 低促性腺功能减退症患者染色体数量是正常的 | 1. Patients with Idiopathic hypogonadotropic hypogonadism (IHH) have a normal number of chromosome. | TRUE |
|  | 2. 低促性腺功能减退症患者的骨龄偏小 | 2. Patients with IHH have reduced bone ages. | TRUE |
|  | 3. 促性腺激素替代治疗对所有低促性腺功能减退症患者都适用 | 3. Gonadotropin replacement therapy is appropriate to all patients with IHH. | TRUE |
| 特发性肺动脉高压（Idiopathic Pulmonary Arterial Hypertension, IPAH） | 1. 确诊肺动脉高压疾病的金标准是右心导管。 | 1. Right heart catheterization is the golden standard for diagnosing Idiopathic pulmonary arterial hypertension (IPAH). | TRUE |
|  | 2. 吸氧对于肺动脉高压患者是便宜又有效的治疗方法，且没有依赖性。 | 2. Oxygen inhalation is an inexpensive and effective treatment without dependency for patients with IPAH. | TRUE |
|  | 3. 无论是哪一类的肺动脉高压，肺（心肺）移植是唯一的治愈的办法。 | 3. Lung (heart and lung) transplantation is the only treatment to cure patients regardless of the type of IPAH. | FALSE |
| 系统性硬化症（Systemic Sclerosis） | 1. 硬皮病主要分为系统性硬皮病和局限型硬皮病两种类型 | 1. Two categories of scleroderma are known: systemic sclerosis (SSc) and localized scleroderma (LoS). | TRUE |
|  | 2. 雷诺现象只在患者暴露于寒冷环境时发生 | 2. Raynaud's phenomenon is triggered only by the exposure to a freezing environment in patients with scleroderma. | FALSE |
|  | 3. mRSS评分系统会选取患者身体16个部位进行皮肤厚硬度评分 | 3. The modified Rodnan Skin Score (mRSS) system estimates the skin thickness and elasticity from 16 body areas in patients with SSc. | TRUE |
| 先天性肾上腺发育不良（Congenital Adrenal Hypoplasia, CAH） | 1. CAH家中出现过患者后，可以通过产前诊断排除患儿出生 | 1. When Congenital adrenal hyperplasia (CAH) is present in families, prenatal testing can assist families to avoid the birth of a child with such disorder. | TRUE |
|  | 2. CAH患者结婚有必要进行婚前基因检测 | 2. It is necessary for patients with CAH to have premarital genetic testing. | TRUE |
|  | 3. 在救治CAH21羟化酶缺乏症失盐新生儿的时候，如果没有盐皮质激素，患儿也可以通过其他方式得到有效治疗 | 3. In the treatment of salt-deprived neonates with CAH due to 21-hydroxylase deficiency, they can also be treated effectively without corticosteroid. | FALSE |
| 血友病（Hemophilia） | 1. 血友病是一种X染色体连锁的先天性出血性疾病 | 1.Hemophilia is a congenital recessive disorder linked to the X-chromosome. | TRUE |
|  | 2. 血友病患者术后应避免肌内注射镇痛剂 | 2. Intramuscular injection of analgesics should be avoided in hemophilia patients after surgery. | TRUE |
|  | 3. 凝血因子替代治疗，目前是唯一有效的血友病治疗方法 | 3. Coagulation factor replacement therapy is the only effective treatment for hemophilia currently. | TRUE |
| 遗传性大疱性表皮松解症（Hereditary Epidermolysis Bullosa, EB） | 1. EB是一种遗传性皮肤病，不具传染性，遗传规律不受性别限制 | 1. Epidermolysis bullosa (EB) is an inherited and noninfectious skin disease without sex limited heredity. | TRUE |
|  | 2. EB有三种主要类型 | 2. There are three main types of EB. | FALSE |
|  | 3. 有过EB患者的家庭如再次怀孕，可以通过产前诊断检测胎儿是否患有EB | 3. Families with a history of EB can test their fetus for EB with a prenatal diagnosis if they conceive again | TRUE |
| 婴儿严重肌阵挛性癫痫（Severe Myoclonic Epilepsy In Infaricy, Dravet syndrome） | 1. 闷热环境、洗热水澡、触摸暖气、电吹风热风都可能诱发Dravet综合征患者癫痫发作 | 1. A sweltering environment, taking a hot bath, touching a heater or a hot hair dryer can trigger seizures in patients with Dravet syndrome. | TRUE |
|  | 2. 多数Dravet综合征患者的致病原因是编码门控钾离子通道的SCN1A基因出现突变 | 2. Most patients with Dravet syndrome are affected by SCN1A gene mutations, which encodes voltage-gated potassium channel. | FALSE |
|  | 3. 当体重为30公斤的患儿出现癫痫持续状态时，可以通过肛门给予地西泮（安定）中止发作，给药量为9至15mg | 3. When an epileptic seizure occurs to a child weighted 30kg, diazepam (diazepam) can be administered through the anus in a dose of 9 to 15 mg. | FALSE |
| 高苯丙氨酸血症（Hyperphenylalaninemia, HPA） | 1. 高苯丙氨酸血症是一种氨基酸代谢异常的常染色体显性遗传疾病 | 1. Hyperphenylalaninemia (HPA) is an autosomal dominant genetic disorder that results in decreased metabolism of the amino acid. | FALSE |
|  | 2. 高苯丙氨酸血症的阶段血值标准监控方式为, 饭后两小时采血 | 2. The standard monitoring method for stage blood value of patients with HPA is to have their blood drawn 2 hours after meal | FALSE |
|  | 3. 若高苯丙氨酸血症患者与健康的正常人结婚生育, 小孩是不会患有此病的 | 3. If a patient with HPA married with a healthy person, their children would not develop the disease. | FALSE |
| 苯丙酮尿症（Phenylketouria, PKU） | 1. 苯丙酮尿症是一种氨基酸代谢异常的常染色体显性遗传疾病 | 1. Phenylketonuria (PKU) is an autosomal dominant genetic disorder with abnormal amino acid metabolism | FALSE |
|  | 2. 苯丙酮尿症患者完全不可以吃阿斯巴甜的食物 | 2. Patients with PKU should not eat food with aspartame at all | TRUE |
|  | 3. 苯丙酮尿症患者每日天然食物和特殊医学奶粉的比例原则是3:8 | 3. The daily ratio of natural food and special medical milk powder is 3:8 for patients with PKU. | FALSE |
| 四氢生物喋呤缺乏症（Tetrahydrobiopterin Deficiency, BH4D） | 1. 四氢生物蝶呤缺乏症患者被医院/医生告知需要终生控制饮食, 特食治疗 | 1. Patients with tetrahydrobiopterin deficiency (BH4D) are informed by the hospital/doctor that they need lifelong diet control and special diet therapy. | FALSE |
|  | 2. 四氢生物蝶呤缺乏症患者的概率占高苯丙氨酸血症患者的10% | 2. BH4D occurred in 10% of patients with hyperphenylalaninemia. | TRUE |
|  | 3. 四氢生物蝶呤缺乏症患者如果与健康的正常人结婚生育，他们的孩子是不会患有此病的 | 3. If a person with BH4D married a healthy person, their children would not develop the disease. | FALSE |
