# Supplementary material for: Physicians’ knowledge on specific rare diseases and its associated factors: a national cross-sectional study from China
Source: Orphanet J Rare Dis. 2022 Mar 5;17:120. doi: 10.1186/s13023-022-02243-7 (PMC8898513; doi:10.1186/s13023-022-02243-7)
Supplement: Supplementary file 2 — Additional file 2. Correct rates of answering each of the three knowledge questions among physicians based on rare diseases. [file 13023_2022_2243_MOESM2_ESM.docx]

**Additional file 2. Correct rates of answering each of the three knowledge questions among physicians based on rare diseases**

| **Type of rare diseases** | **Correct rates of answering each question, N(%)** | | |
| --- | --- | --- | --- |
|  | **Q1** | **Q2** | **Q3** |
| Albinism | 337(72.3%) | 326(70.0%) | 170(36.5%) |
| Osteogenesis Imperfecta | 82(69.5%) | 55(46.6%) | 40(33.9%) |
| Homozygous Hypercholesterolemia | 23(92.0%) | 14(56.0%) | 15(60.0%) |
| Duchenne Muscular Dystrophy | 24(35.8%) | 50(74.6%) | 53(79.1%) |
| Multiple Sclerosis | 299(86.2%) | 82(23.6%) | 54(15.6%) |
| Fabry Disease | 13(65.0%) | 17(85.0%) | 11(55.0%) |
| Hepatolenticular Degeneration | 344(90.8%) | 288(76.0%) | 188(49.6%) |
| Gaucher Disease | 12(75.0%) | 9(56.3%) | 10(62.5%) |
| Huntington Disease | 17(100.0%) | 3(17.6%) | 10(58.8%) |
| Amyotrophic Lateral Sclerosis | 19(15.3%) | 55(44.4%) | 120(96.8%) |
| Spinocerebellar Ataxia | 4(26.7%) | 10(66.7%) | 3(20.0%) |
| Spinal Muscular Atrophy | 24(80.0%) | 16(53.3%) | 2(6.7%) |
| Spinal and Bulbar Muscular Atrophy | 2(50.0%) | 0(0.0%) | 1(25.0%) |
| Tuberous Sclerosis Complex | 36(94.7%) | 1(2.6%) | 32(84.2%) |
| Kallmann Syndrome | 14(53.8%) | 24(92.3%) | 9(34.6%) |
| Langerhans Cell Histiocytosis | 106(96.4%) | 72(65.5%) | 85(77.3%) |
| Lymphangioleiomyomatosis | 45(78.9%) | 39(68.4%) | 11(19.3%) |
| Marfan Syndrome | 217(96.9%) | 181(80.8%) | 17(7.6%) |
| Niemann-Pick Disease | 10(83.3%) | 11(91.7%) | 9(75.0%) |
| Mucopolysaccharidosis | 6(100.0%) | 3(50.0%) | 5(83.3%) |
| Prader-Willi Syndrome | 18(39.1%) | 31(67.4%) | 9(19.6%) |
| General Myathenic Gravis | 78(53.4%) | 103(70.5%) | 91(62.3%) |
| Neuromyelitis Optica | 83(93.3%) | 71(79.8%) | 23(25.8%) |
| Type II Glycogen Storage Disease | 3(100.0%) | 1(33.3%) | 0(0.0%) |
| Idiopathic Hypogonadotropic Hypogonadism | 29(59.2%) | 40(81.6%) | 14(28.6%) |
| Idiopathic Pulmonary Arterial Hypertension | 159(94.6%) | 118(70.2%) | 104(61.9%) |
| Systemic Sclerosis | 135(88.8%) | 120(78.9%) | 93(61.2%) |
| Congenital Adrenal Hypoplasia | 33(75.0%) | 41(93.2%) | 15(34.1%) |
| Hemophilia | 246(84.0%) | 225(76.8%) | 240(81.9%) |
| Hereditary Epidermolysis Bullosa | 17(73.9%) | 2(8.7%) | 14(60.9%) |
| Severe Myoclonic Epilepsy In Infaricy | 4(66.7%) | 1(16.7%) | 3(50.0%) |
| Hyperphenylalaninemia | 3(60.0%) | 1(20.0%) | 5(100.0%) |
| Phenylketonuria | 13(18.3%) | 23(32.4%) | 11(15.5%) |
| Tetrahydrobiopterin Deficiency | 0(0.0%) | 0(0.0%) | 1(100.0%) |
