# Supplementary material for: Physicians’ knowledge on specific rare diseases and its associated factors: a national cross-sectional study from China
Source: Orphanet J Rare Dis. 2022 Mar 5;17:120. doi: 10.1186/s13023-022-02243-7 (PMC8898513; doi:10.1186/s13023-022-02243-7)
Supplement: Supplementary file 3 — Additional file 3. Frequencies of physicians in different specialties based on 34 rare diseases of concern. [file 13023_2022_2243_MOESM3_ESM.docx]

**Additional file 3. Frequencies of physicians in different specialties based on 34 rare diseases of concern**

| **Disease Code**  **Specialties** | **1** | **2** | **3** | **4** | **5** | **6** | **7** | **8** | **9** | **10** | **11** | **12** | **13** | **14** | **15** | **16** | **17** | **18** | **19** | **20** | **21** | **22** | **23** | **24** | **25** | **26** | **27** | **28** | **29** | **30** | **31** | **32** | **33** | **34** | **No. of diseases** | **No. of physicians** |
| --- | --- | --- | --- | --- | --- | --- | --- | --- | --- | --- | --- | --- | --- | --- | --- | --- | --- | --- | --- | --- | --- | --- | --- | --- | --- | --- | --- | --- | --- | --- | --- | --- | --- | --- | --- | --- |
| Internal medicine | 76 | 12 | 19 | 23 | 186 | 17 | 165 | 11 | 4 | 76 | 5 | 9 | 0 | 9 | 21 | 50 | 42 | 76 | 7 | 3 | 13 | 52 | 51 | 3 | 43 | 124 | 87 | 23 | 145 | 3 | 2 | 0 | 12 | 1 | 32 | 1370 |
| Anesthesiology | 13 | 0 | 0 | 0 | 2 | 0 | 0 | 0 | 0 | 0 | 1 | 0 | 0 | 0 | 0 | 0 | 0 | 4 | 0 | 0 | 0 | 8 | 0 | 0 | 0 | 0 | 1 | 0 | 4 | 0 | 0 | 0 | 0 | 0 | 7 | 33 |
| Dermatology | 19 | 0 | 0 | 0 | 1 | 0 | 0 | 0 | 0 | 0 | 0 | 0 | 0 | 9 | 0 | 3 | 0 | 1 | 0 | 0 | 0 | 0 | 0 | 0 | 0 | 0 | 20 | 0 | 0 | 4 | 0 | 0 | 0 | 0 | 7 | 57 |
| Emergency medicine | 19 | 3 | 0 | 1 | 9 | 0 | 5 | 0 | 4 | 3 | 0 | 0 | 0 | 0 | 0 | 0 | 0 | 10 | 0 | 0 | 0 | 11 | 1 | 0 | 0 | 6 | 2 | 0 | 13 | 1 | 0 | 0 | 1 | 0 | 15 | 89 |
| Infectious dept | 5 | 1 | 0 | 0 | 1 | 0 | 52 | 0 | 0 | 1 | 0 | 0 | 0 | 0 | 0 | 0 | 0 | 4 | 0 | 0 | 0 | 2 | 0 | 0 | 0 | 0 | 1 | 0 | 1 | 0 | 0 | 0 | 1 | 0 | 10 | 69 |
| Intensive care unit | 13 | 3 | 0 | 0 | 10 | 0 | 5 | 1 | 0 | 9 | 0 | 0 | 1 | 0 | 0 | 0 | 1 | 7 | 0 | 0 | 1 | 17 | 1 | 0 | 0 | 8 | 2 | 0 | 7 | 0 | 0 | 0 | 0 | 0 | 15 | 86 |
| Laboratory dept | 2 | 1 | 0 | 0 | 0 | 0 | 0 | 0 | 0 | 0 | 0 | 0 | 0 | 0 | 0 | 0 | 0 | 0 | 0 | 0 | 0 | 0 | 0 | 0 | 0 | 0 | 0 | 0 | 0 | 0 | 0 | 0 | 0 | 0 | 2 | 3 |
| Obstetrics and gynecology | 46 | 2 | 0 | 2 | 4 | 0 | 10 | 0 | 0 | 2 | 1 | 3 | 0 | 1 | 1 | 0 | 3 | 15 | 0 | 0 | 0 | 3 | 0 | 0 | 4 | 1 | 2 | 1 | 17 | 0 | 0 | 0 | 15 | 0 | 19 | 133 |
| Oncology | 21 | 1 | 1 | 0 | 6 | 0 | 3 | 0 | 0 | 2 | 0 | 0 | 0 | 0 | 0 | 10 | 1 | 2 | 0 | 0 | 0 | 6 | 0 | 0 | 0 | 5 | 4 | 1 | 9 | 0 | 0 | 0 | 2 | 0 | 15 | 74 |
| Ophthalmology | 24 | 0 | 0 | 0 | 1 | 0 | 3 | 0 | 1 | 1 | 0 | 0 | 0 | 0 | 0 | 1 | 0 | 16 | 0 | 0 | 0 | 3 | 19 | 0 | 0 | 0 | 0 | 0 | 0 | 0 | 0 | 0 | 0 | 0 | 9 | 69 |
| Orthopedic surgery, medical cosmetology | 6 | 0 | 0 | 0 | 0 | 0 | 0 | 0 | 0 | 0 | 0 | 0 | 0 | 0 | 0 | 1 | 0 | 0 | 0 | 0 | 0 | 0 | 0 | 0 | 0 | 0 | 0 | 0 | 2 | 1 | 0 | 0 | 1 | 0 | 5 | 11 |
| Otolaryngology | 13 | 0 | 0 | 0 | 2 | 0 | 2 | 0 | 1 | 0 | 0 | 0 | 0 | 1 | 0 | 3 | 0 | 4 | 0 | 0 | 0 | 5 | 0 | 0 | 0 | 0 | 0 | 0 | 6 | 0 | 0 | 0 | 0 | 0 | 9 | 37 |
| Pain medicine | 4 | 0 | 0 | 0 | 0 | 0 | 0 | 0 | 0 | 0 | 0 | 0 | 0 | 0 | 0 | 0 | 0 | 2 | 0 | 0 | 0 | 0 | 0 | 0 | 0 | 0 | 0 | 0 | 0 | 0 | 0 | 0 | 0 | 0 | 2 | 6 |
| Pathology | 0 | 1 | 0 | 0 | 0 | 0 | 0 | 0 | 0 | 0 | 0 | 0 | 0 | 1 | 0 | 6 | 0 | 0 | 0 | 0 | 0 | 0 | 0 | 0 | 0 | 0 | 0 | 0 | 0 | 0 | 0 | 0 | 0 | 0 | 3 | 8 |
| Pediatrics | 32 | 20 | 0 | 29 | 9 | 0 | 63 | 3 | 0 | 0 | 0 | 6 | 0 | 4 | 0 | 12 | 0 | 8 | 3 | 3 | 31 | 4 | 0 | 0 | 0 | 10 | 9 | 17 | 23 | 9 | 2 | 3 | 24 | 0 | 22 | 324 |
| Psychiatry | 3 | 0 | 0 | 0 | 5 | 0 | 7 | 0 | 0 | 1 | 0 | 0 | 0 | 0 | 0 | 0 | 0 | 0 | 0 | 0 | 0 | 1 | 0 | 0 | 0 | 0 | 0 | 0 | 0 | 0 | 0 | 0 | 0 | 0 | 5 | 17 |
| Radiology | 25 | 16 | 1 | 0 | 27 | 1 | 15 | 0 | 0 | 2 | 0 | 1 | 0 | 5 | 0 | 6 | 4 | 16 | 0 | 0 | 0 | 0 | 3 | 0 | 0 | 3 | 0 | 0 | 7 | 1 | 0 | 0 | 5 | 0 | 17 | 138 |
| Sports medicine, rehabilitation | 13 | 3 | 1 | 4 | 22 | 0 | 4 | 0 | 1 | 5 | 0 | 8 | 1 | 1 | 0 | 0 | 0 | 1 | 1 | 0 | 1 | 2 | 9 | 0 | 0 | 1 | 4 | 0 | 2 | 0 | 1 | 0 | 1 | 0 | 21 | 86 |
| Stomatology | 10 | 0 | 0 | 0 | 0 | 1 | 0 | 0 | 0 | 0 | 1 | 0 | 0 | 0 | 0 | 8 | 0 | 1 | 0 | 0 | 0 | 1 | 0 | 0 | 0 | 0 | 1 | 0 | 6 | 0 | 0 | 0 | 0 | 0 | 8 | 29 |
| Surgery | 93 | 52 | 1 | 4 | 34 | 1 | 36 | 1 | 6 | 17 | 5 | 1 | 2 | 7 | 4 | 10 | 4 | 52 | 0 | 0 | 0 | 21 | 1 | 0 | 2 | 5 | 5 | 2 | 37 | 4 | 1 | 1 | 6 | 0 | 29 | 415 |
| Traditional Chinese Medicine | 8 | 2 | 1 | 0 | 13 | 0 | 1 | 0 | 0 | 1 | 1 | 1 | 0 | 0 | 0 | 0 | 0 | 0 | 0 | 0 | 0 | 1 | 1 | 0 | 0 | 0 | 5 | 0 | 3 | 0 | 0 | 0 | 1 | 0 | 13 | 39 |
| Others | 21 | 1 | 1 | 4 | 15 | 0 | 8 | 0 | 0 | 4 | 1 | 1 | 0 | 0 | 0 | 0 | 2 | 5 | 1 | 0 | 0 | 9 | 3 | 0 | 0 | 5 | 9 | 0 | 11 | 0 | 0 | 1 | 2 | 0 | 19 | 104 |

1=Albinism

2=Osteogenesis Imperfecta

3=Homozygous Hypercholesterolemia

4=Duchenne Muscular Dystrophy

5=Multiple Sclerosis

6=Fabry Disease

7=Hepatolenticular Degeneration

8=Gaucher＇s Disease

9=Huntington Disease

10=Amyotrophic Lateral Sclerosis

11=Spinocerebellar Ataxia

12=Spinal Muscular Atrophy

13=Spinal and Bulbar Muscular Atrophy

14=Tuberous Sclerosis Complex

15=Kallmann Syndrome

16=Langerhans Cell Histiocytosis

17=Lymphangioleiomyomatosis

18=Marfan Syndrome

19=Niemann-Pick Disease

20=Mucopolysaccharidosis

21=Prader-Willi Syndrome

22=General Myathenic Gravis

23=Neuromyelitis Optica

24=Type II Glycogen Storage Disease

25=Idiopathic Hypogonadotropic Hypogonadism

26=Idiopathic Pulmonary Arterial Hypertension

27=Systemic Sclerosis

28=Congenital Adrenal Hypoplasia

29=Hemophilia

30=Hereditary Epidermolysis Bullosa

31=Severe Myoclonic Epilepsy In Infaricy

32=Hyperphenylalaninemia

33=Phenylketouria

34=Tetrahydrobiopterin Deficiency
